# School-age structural and functional MRI and lung function in children following lung resection for congenital lung malformation in infancy

**DOI:** 10.1007/s00247-022-05317-7

**Published:** 2022-03-19

**Authors:** Corin Willers, Lukas Maager, Grzegorz Bauman, Dietmar Cholewa, Enno Stranzinger, Luigi Raio, Carmen Casaulta, Philipp Latzin

**Affiliations:** 1 Division of Pediatric Respiratory Medicine, Department of Pediatrics, Inselspital, Bern University Hospital, University of Bern, Freiburgstrasse 8, 3010 Bern, Switzerland; 2 Division of Radiological Physics, Department of Radiology, University of Basel Hospital, Basel, Switzerland; 3 Department of Biomedical Engineering, University of Basel, Allschwil, Switzerland; 4 Department of Pediatric Surgery, Inselspital, Bern University Hospital, University of Bern, Bern, Switzerland; 5 Institute of Diagnostic, Interventional and Pediatric Radiology, Inselspital, Bern University Hospital, University of Bern, Bern, Switzerland; 6 Department of Obstetrics and Gynecology, Inselspital, Bern University Hospital, University of Bern, Bern, Switzerland

**Keywords:** Children, Congenital, Infants, Lung, Magnetic resonance imaging, Malformation, Pulmonary, Surgery

## Abstract

**Background:**

The management of asymptomatic congenital lung malformations is debated. Particularly, there is a lack of information regarding long-term growth and development of the remaining lung in children following lung resection for congenital lung malformations. In addition to conventional pulmonary function tests, we used novel functional magnetic resonance imaging (MRI) methods to measure perfusion and ventilation.

**Objective:**

To assess functionality of the remaining lung expanded into the thoracic cavity after resection of congenital lung malformations.

**Materials and methods:**

A prospective, cross-sectional pilot study in five children who had surgery for congenital lung malformations during infancy. Participants had structural and functional MRI as well as spirometry, body plethysmography and multiple breath washout at school age.

**Results:**

Structural MRI showed an expansion of the remaining lung in all cases. Fractional ventilation and relative perfusion of the expanded lung were locally decreased in functional MRI. In all other parts of the lungs, fractional ventilation and relative perfusion were normal in all children. There was an association between overall impairment of perfusion and elevated lung clearance index. The results of spirometry and body plethysmography varied between patients, including normal lung function, restriction and obstruction.

**Conclusion:**

Fractional ventilation and relative perfusion maps from functional MRI specifically locate impairment of the remaining lung after lung resection. These changes are not captured by conventional measures such as structural MRI and standard pulmonary function tests. Therefore, following lung resection for congenital lung malformation, children should be investigated more systematically with functional lung MRI.

**Supplementary Information:**

The online version contains supplementary material available at 10.1007/s00247-022-05317-7.

## Introduction

Congenital lung malformations, including congenital pulmonary airway malformation (CPAM), extra- and intralobar bronchopulmonary sequestration, congenital lobar and segmental emphysema, and bronchogenic cyst, are amongst the most frequent abnormalities detected in utero [1, 2]. With improved prenatal ultrasound, the incidence has seen a rise; however, congenital lung malformations remain a rare condition in the overall population [3, 4].To date, the management of congenital lung malformations, such as CPAM, bronchopulmonary sequestration and the overlap of these two (hybrid lesions), is still controversial. For children symptomatic at birth, a surgical approach, such as lobectomy, segmentectomy or cyst removal, is widely accepted [5, 6]. However, the management of asymptomatic congenital lung malformations is controversial. Early elective resection of the malformations aims to prevent complications such as recurrent pulmonary infection, pneumothorax or malignancy. Early intervention is supported by the idea of compensatory growth of the remaining lung parenchyma after lung resection, resulting in physiological lung growth [7–9]. However, it is still debated if the lung is capable of compensatory growth or primarily extends into the cavity. In case of compensatory growth, studies have suspected a potential for a structure-function dissociation, with an unequal growth of conducting airways and blood vessels in comparison to acinar tissue [10]. It is especially unclear whether the expansion or growth of the lung results in balanced ventilation and perfusion. Long-term evolution of lung function as measured by conventional lung function tests is therefore controversial [11–13]. The aim of this pilot study was to assess functionality of expanded lung tissue, using lung function tests and novel magnetic resonance imaging (MRI)-based functional methods to measure ventilation and perfusion.

## Materials and methods

For this cross-sectional, single-center pilot study, we searched the local picture archiving and communication and hospital information systems for reports mentioning congenital lung malformation, CPAM, bronchopulmonary sequestration, congenital emphysema or bronchogenic cyst as indications for imaging and as differential diagnosis or diagnosis in patients born between the years 2008 and 2016. Inclusion criteria were (1) a postnatal, surgical malformation resection, with histologically confirmed congenital lung malformation, (2) age between 4 and 18 years, and (3) the ability to perform pulmonary function testing and to tolerate a non-sedated MRI. The exclusion criterion was an isolated extrathoracic malformation. The study was approved by the local Ethics Committee, and parents or caregivers provided written informed consent. Multiple breath washout, fractional exhaled nitric oxide, body plethysmography and spirometry and structural and functional lung MRI were performed in order on the same day.

The multiple breath washout measurement was performed using an unmodified device according to international guidelines (Exhalyzer D; Eco Medics AG, Duernten, Switzerland) [14]. The multiple breath washout test and its main outcome, the lung clearance index, assess the overall ventilation homogeneity based on gas distribution in large and small airways. The upper limit of normality was set at 7.9 turnovers [15]. Fractional exhaled nitric oxide testing was performed according to international guidelines [16]. It is a measure of eosinophilic airway inflammation resulting from allergic asthma (among other causes). Spirometry and body plethysmography (Jaeger MasterScreen; CareFusion, Hochberg, Germany) were performed according to international guidelines [17]. The main outcomes were the dynamic volumes, such as forced vital capacity (FVC) and forced expiratory volume in one second (FEV1) and the lower limit of normality was set at −1.64 z-scores for spirometry. The main outcomes for body plethysmography tests were static volumes, such as the quotient of total lung capacity to residual volume and the functional residual capacity, which represent markers of hyperinflation. Spirometry outcomes assess the extent of ventilatory obstruction and restriction in the large airways.

Imaging was performed on a 1.5-T whole-body MRI scanner (MAGNETOM Aera; Siemens Healthineers, Erlangen, Germany) while the children were awake and not sedated. No intravenous contrast agent was used. Parents or caregivers were allowed in the cabin to accompany the participant if necessary. The structural protocol consisted of axial and coronal T2-weighted single-shot fast spin echo with half-Fourier acquisition in multiple inspiratory breath hold (repetition time [TR]/echo time [TE]: 1,200/92 ms; slice thickness: 5 mm), coronal T2-weighted fast spin echo with fat saturation and a respiratory trigger (TR/TE: >2,500/76 ms; slice thickness: 5 mm), coronal T1/T2-weighted 3-D ultra-fast steady-state free precession in inspiratory breath hold (TR/TE: 1.5/0.7 ms; slice thickness: 2 mm), axial T1-weighted 3-D spoiled gradient echo with parallel imaging in one inspiratory breath hold (TR/TE: 6.8/2.4 ms; slice thickness: 4 mm) and sagittal T2-weighted balanced steady-state free precession (TR/TE: 3.3/1.3 ms; slice thickness: 5 mm) sequence [18, 19]. The reader (E.S.) is a pediatric radiologist with >5 years of experience in chest MRI.

Functional imaging was performed with noninvasive, free-breathing, proton-based matrix pencil decomposition MRI (MP-MRI), assessing perfusion and ventilation of the lung simultaneously [20]. First, patients were scanned with a custom ultra-fast steady-state free precession pulse sequence to obtain time-resolved 2-D image series of the lungs. Images were acquired at multiple coronal slice positions to cover the whole lung. Pulse sequence parameters were as follows: field of view (FOV)=450×450 mm^2^, matrix size=128×128, TE/TR=0.67/1.46 ms, flip angle=60°, slice thickness=12 mm, bandwidth=2,056 Hz/voxel, acquisition time per image=110 ms, acquisition rate=3.3 images/s, acquisition time per slice=50 s, generalized autocalibrating partial parallel acquisition (GRAPPA) factor=2 and predefined default shim settings (tune-up) [21]. Afterward, a dedicated image registration algorithm was applied to align lung structure on a baseline image in mid respiratory state (baseline image) [22]. From the baseline image, a neural network was applied to segment the lung parenchyma with exclusion of the main vessels [23]. The technique uses periodic variations of regional signal intensity in the lung parenchyma caused by breathing and blood flow. The amplitudes of those physiological processes can be spectrally retrieved using matrix pencil decomposition.

The matrix pencil algorithm is used to calculate maps of fractional ventilation and relative perfusion [20]. To describe overall functional impairment of the whole lung, we determined percentage of the lung volume with impaired or defected ventilation (VDP) and defected perfusion (QDP) as the main outcomes from MP-MRI. To calculate VDP and QDP, we analyzed the signal intensity histogram by performing thresholding, where all voxels below 70% of the median value were defined as defects [24, 25]. To specifically localize regional defects, we further analyzed the functional maps qualitatively. As atypical or reduced diaphragmatic motion potentially interferes with fractional ventilation analysis, we qualitatively analysed the diaphragmatic motion in the time-resolved 2-D ultra-fast steady-state free precession pulse sequence.

Fourier decomposition based proton MRI and its successor MP-MRI are validated against single-photon emission computed tomography (SPECT-CT), dynamic contrast enhanced and helium-3 hyperpolarized MRI [24, 26, 27]. In previous studies, VDP and QDP correlated well with lung function parameters, such as lung clearance index and FEV1 in pediatric patients with cystic fibrosis [25]. From previous studies in same aged, pediatric healthy controls an upper limit of normality was estimated for VDP at <25% and QDP <21% [25, 28]. The overall scan duration was 25 to 40 min.

## Results

From an initial 73 screened patients, 13 met the inclusion criteria and were contacted for study participation. Finally, five patients were enrolled. A flow diagram of the study enrollment is in Online Supplementary Material 1. The FEV1 was abnormal in three of the patients and three had an abnormal lung clearance index, while three patients had increased perfusion defects, and none had ventilation defects. A summary of the five cases, including detailed pulmonary function test results, is in Table 1. Apart from a potential reduction in the validity of the fractional ventilation maps, there was no atypical or severely reduced diaphragmatic motion demonstrated.

**Table 1:** Clinical data and results of functional magnetic resonance imaging (MRI), multiple breath washout (MBW), pulmonary function tests (spirometry, body plethysmography) and fraction of exhaled nitric oxide (FeNO)

	Case 1	Case 2	Case 3	Case 4	Case 5
Sex, m/f	f	m	f	m	f
Gestational age at diagnosis, weeks	21 3/7	24 0/7	34 1/7	25 0/7	36 2/7
SGA/IGUR	no	no	no	no	no
Gestational age at birth, weeks	41 0/7	40 1/7	35 0/7	35 2/7	40 3/7
Age at surgery, weeks	66 6/7	18 1/7	1 1/7	0 5/7	1 1/7
Weight at surgery, kg	–	5.92	3.05	2.2	3.37
Site of resected lung size cm x cm x cm	Right lower lobe	Right lower lobe BPS, 4× 2× 0.5	Right lower lobe	Left lower lobe	Segments I, II, III left
Type of lung resection surgery	Thoracotomy: Lobectomy right lower lobe	Thoracotomy: Lobectomy right lower lobe, resection BPS	Thoracotomy: Lobectomy right lower lobe	Thoracotomy: Lobectomy left lower lobe	Thoracotomy: Atypical stapler-resection, seg I-III left
Type of lung malformation	Hybrid lesion (CPAM type 1, intralobar BPS)	Hybrid lesion (CPAM type 2, extrathoracic BPS)	CPAM type 1	CPAM type 1	CPAM type 1
Syndromal, y/n	n	y	y	n	n
Comorbidity	–	Small diaphragmatic hernia right, diaphragmatic elevation right	Pectus excavatum, incomplete prune belly syndrome	–	–
Age at examination, years	7 4/12	5 3/12	10 4/12	6 10/12	6 10/12
Functional MRI					
Ventilation defect percentage, VDP (norm <25%)	13.14	13.60	22.28	13.40	13.60
Perfusion defect percentage, QDP (norm <21%)	26.13	24.52	22.76	18.29	14.46
LCI 2.5% (norm 7.9 turnover)	8.37	8.4	8.23	7.32	6.61
Spirometry					
FVC, %pred (z-score)	110 (0.79)	90 (−0.76)	63 (−3.22)	72 (−2.28)	112 (0.92)
FEV1, %pred (z-score)	97 (−0.27)	82 (−1.38)	57 (−3.58)	62 (−2.98)	105 (0.41)
FEV1(%)/FVC(%), %	80	83	79	77	84
Body plethysmography					
FRC, %pred	117	111	47	77	90
TLC, %pred	98	95	62	74	98
RV, %pred	95	145	65	124	117
RV(L)/TLC(L), %	26.3	42.3	27.2	44.5	31.4
FeNO, ppb (norm <20)	4.7	4.3	7.1	5.9	10.3

Values considered as normal: functional MRI (ventilation defect percentage [VDP] <25%, perfusion defect percentage [QDP] <21%), MBW (LCI: <7.9 TO), spirometry (FVC, FEV1: ≥80% of predicted value; FEV1%/FVC%: ≥80%), body plethysmography (TLC: ≥80% of predicted value, RV: ≤ 135% of predicted value, RV%/TLC%: ≤ 35%), FeNO (<20 pbb)

*BPS* bronchopulmonary sequestration, *CPAM* congenital pulmonary airway malformation, *f* female, *FEV1* forced expiratory volume in the first second, *FVC* forced vital capacity*, IGUR* intrauterine growth restriction, *L* litre, *LCI* lung clearance index, *m* male, *n* no, *ppb* parts per billion, *pred* predicted, *RV* residual volume, *SGA* small for gestational age, *TLC* total lung capacity, *y* yes

### Case 1

A 7-year-old girl with a hybrid lesion in the right lower lobe, consisting of an intralobar bronchopulmonary sequestration with an associated CPAM type 1 (Stocker), first suspected at 21 weeks’ gestational age. A lobectomy of the right lower lobe was performed at 15 postnatal months; diagnosis was confirmed by histopathology. She had no other comorbidities and an uneventful clinical history up to the time of recruitment.

**Fig. 1:**
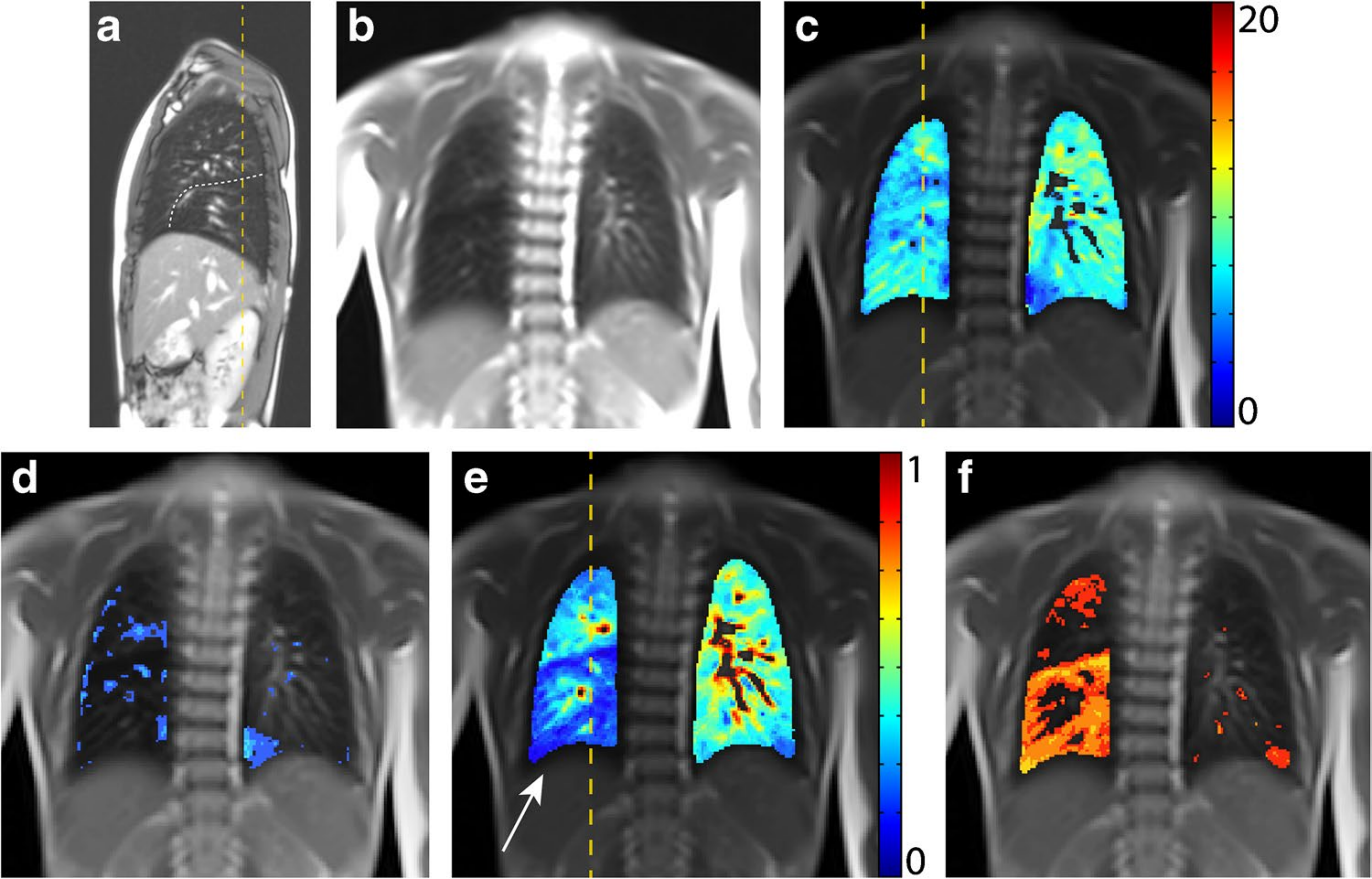
A 7-year-old girl following lobectomy of the right lower lobe for hybrid lesion (intralobar bronchopulmonary sequestration, CPAM type 1) and an increased lung clearance index of 8.37 turnovers. **a** A sagittal T2-weighted image (repetition time [TR]/echo time [TE] 3.3/1.3 ms) and (**b)** a coronal T1/2-weighted image (TR/TE 1.5/07 ms) demonstrate expansion of the middle lobe into the cavity of the resected right lower lobe. **c-f** Coronal functional magnetic resonance imaging maps: **(c)** coronal fractional ventilation, **(d)** fractional ventilation defect percentage (brighter blue areas represent reduced function), **(e)** relative perfusion and (**f)** relative perfusion defect maps (brighter orange areas represent reduced function) show a fractional ventilation defect percentage (13.1%) within the norm and an increased perfusion defect percentage (26.1%) in the area of the former right lower lobe, to which region the middle lobe has expanded **(e)** (*white arrow*). *Yellow dashed lines* indicate plane levels. *White dashed line* indicates lung fissure for better visibility. *CPAM* congenital pulmonary airway malformation

Current structural MRI shows a slightly hyperinflated middle lobe, which has expanded to the right lower posterior thorax. In this area, functional MRI shows locally reduced fractional ventilation and a large local decrease of perfusion, which leads to an increased overall QDP (Fig. 1). Overall (global) fractional VDP, however, is normal. Body plethysmography and spirometry (Table 1) are normal, excluding a severe restriction, obstruction or hyperinflation. The lung clearance index is above the norm, indicating inhomogeneity of ventilation.

### Case 2

A 5-year-old boy with a hybrid lesion, consisting of a CPAM type 2 in the right lower lobe and an extralobar and extrathoracic bronchopulmonary sequestration on the right, first diagnosed at 24 weeks’ gestational age. He also had a small diaphragmatic hernia and an elevated diaphragm on the right. A lobectomy of the right lower lobe and resection of the bronchopulmonary sequestration were performed at 4 months’ postnatal age. An uneventful course after surgery up to the present is reported.

**Fig. 2:**
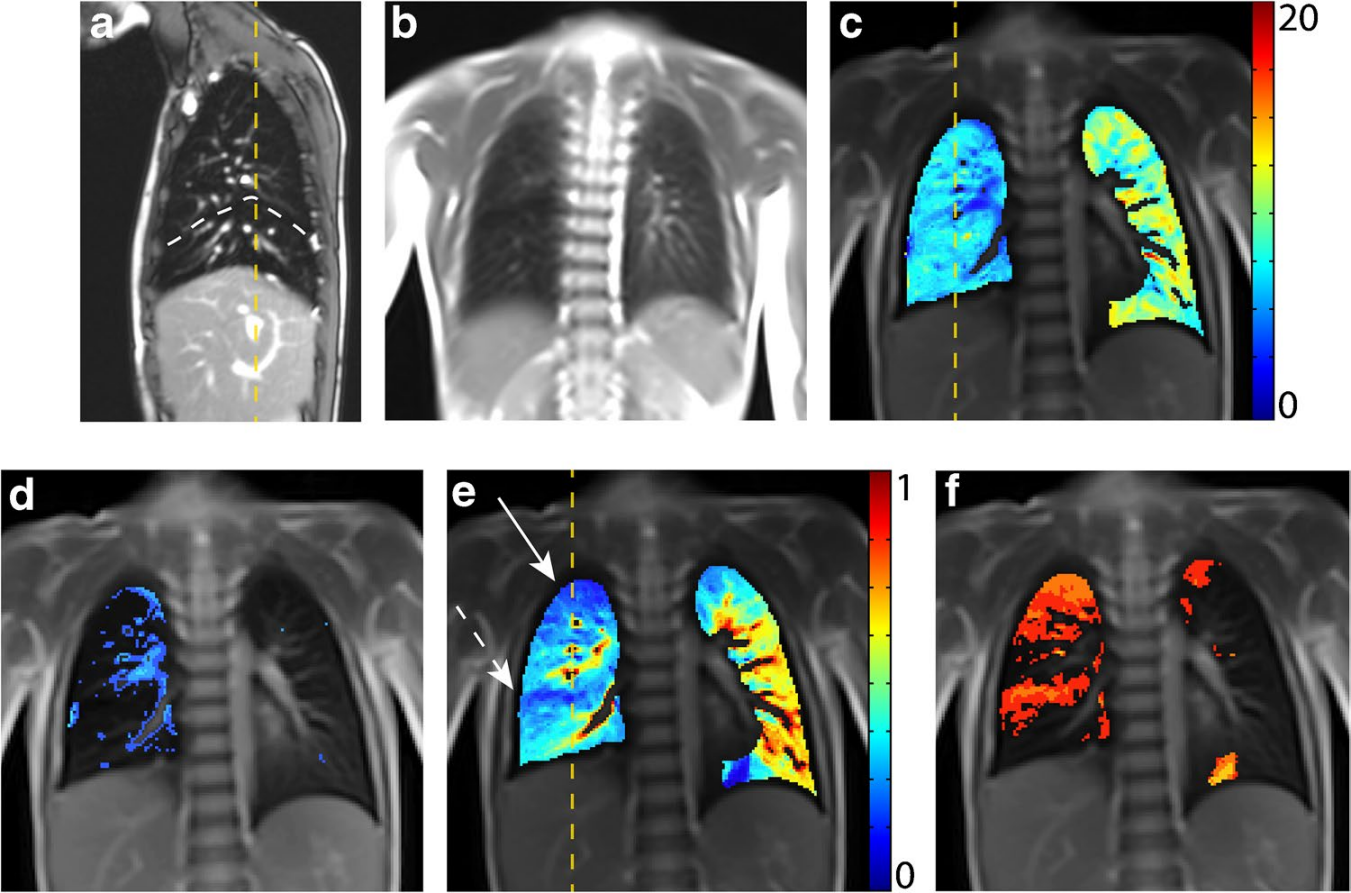
A 5-year-old boy following lobectomy of the right lower lobe for CPAM type 2 and resection of (**a)** subdiaphragmatic bronchopulmonary sequestration on the right side with an increased lung clearance index of 8.4 turnovers. A sagittal T2-weighted image (**a**) (repetition time [TR]/echo time [TE] 3.3/1.3 ms) and **(b)** coronal T1/2-weighted image (TR/TE 1.5/07 ms) demonstrate an expansion of the residual right lung into the lower right thoracic cavity. **c-f** Coronal functional magnetic resonance imaging maps: **(c)** coronal fractional ventilation, **(d)** fractional ventilation defect percentage (brighter blue areas represent reduced function), **(e)** relative perfusion and (**f)** relative perfusion defect maps (brighter orange areas represent reduced function) show a fractional ventilation defect percentage (13.6%) within the norm and an increased perfusion defect percentage (24.5%) predominantly in the area of the right middle (*dashed arrow*) and upper lobe (*solid arrow*). *Yellow dashed lines* indicate plane levels. *White dashed* line indicates lung fissure for better visibility. *CPAM* congenital pulmonary airway malformation

On the structural MRI, a smaller right lung volume, an elevated diaphragm and decreased motility of the right diaphragm are seen. The middle lobe has expanded into the thoracic cavity of the former right lower lobe. In the area of the expanded middle lobe and of the upper lobe, functional MRI shows locally impaired fractional ventilation and perfusion (Fig. 2). This leads to an increased overall QDP but a normal overall VDP. The lung clearance index is increased.

### Case 3

A 10-year-old girl with a CPAM type 1 in the right lower lobe, first diagnosed at 34 weeks’ gestational age. A lobectomy of the right lower lobe was performed 8 days after birth. Additionally, pectus excavatum and incomplete prune belly syndrome became notable. The postoperative course was uneventful. At present, decreased physical performance and difficulties in breathing during physical exertion are reported.

**Fig. 3:**
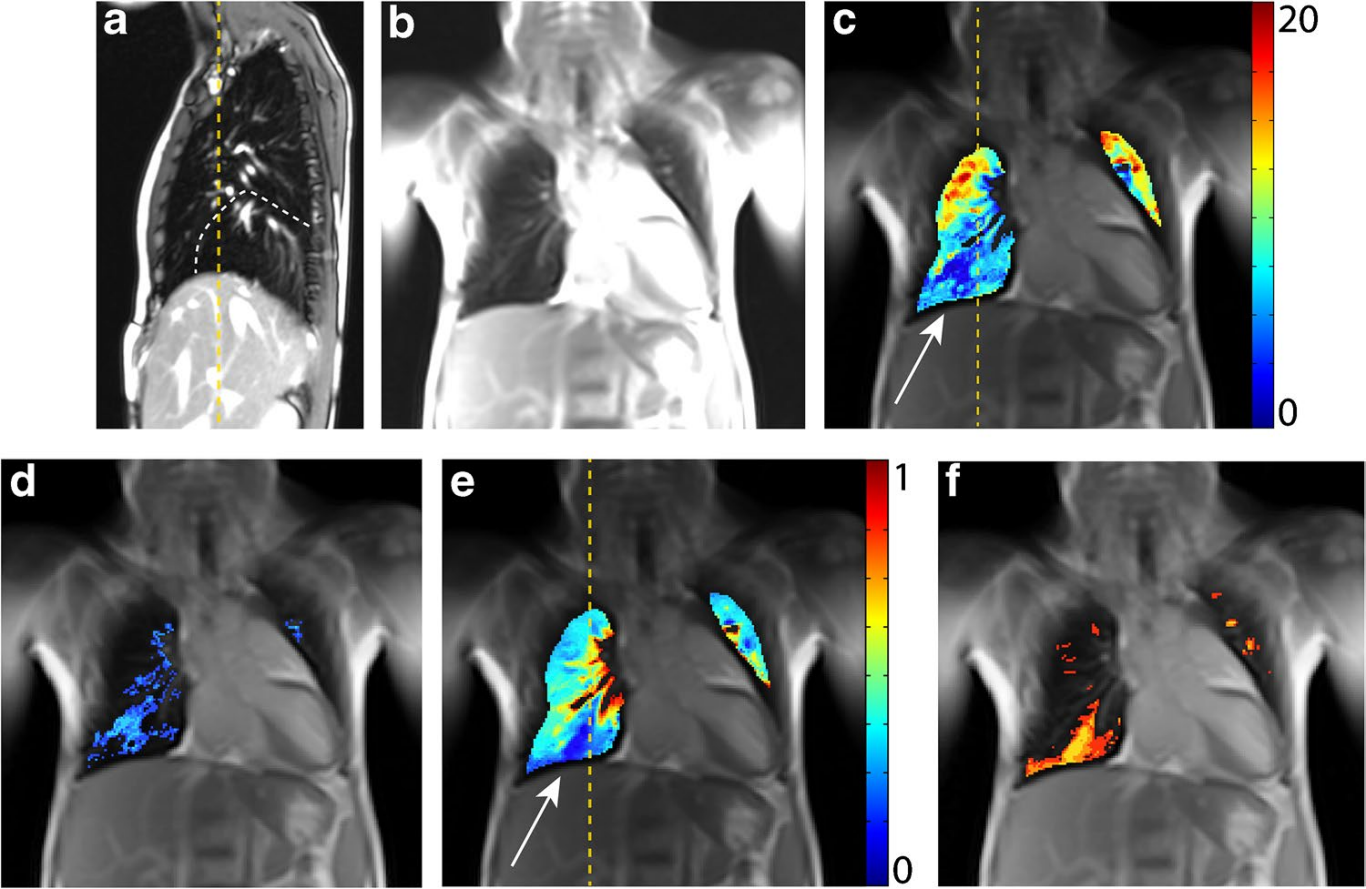
A 10-year-old girl following lobectomy of the right lower lobe for CPAM type 1 with an increased lung clearance index of 8.23 turnovers. A sagittal T2-weighted image (**a**) (repetition time [TR]/echo time [TE] 3.3/1.3 ms) and (**b**) coronal T1/2-weighted image (TR/TE 1.5/07 ms) demonstrate that the middle lobe replaces the area of the former right lower lobe. **c-f** Coronal functional magnetic resonance imaging maps: (**c**) fractional ventilation, (**d**) fractional ventilation defect percentage (brighter blue areas represent reduced function), **(e)** relative perfusion and (**f)** relative perfusion defect maps (brighter orange areas represent reduced function) show a local reduced fractional ventilation (**c**), while the overall fractional ventilation defect percentage is within the norm and an increased perfusion defect percentage (22.8%) predominantly in the area of the right middle lobe (**e**) (*white arrow*). *Yellow dashed lines* indicate plane levels. *CPAM* congenital pulmonary airway malformation

The middle lobe replaces the area of the former right lower lobe as demonstrated by the structural MRI. From the time-resolved 2-D sequences, hyperinflation of the right upper lobe is suspected. Correspondingly, the fractional ventilation is slightly reduced in the same area, but it is not so severe that this area is quantified as having a fractional ventilation defect. Furthermore, an increased overall perfusion impairment is noted, mainly which is mainly localized in the lower right thoracic cavity, which contains the expanded middle lobe (Fig. 3). Pulmonary function tests show restriction and an increased lung clearance index.

### Case 4

A 6-year-old boy with a CPAM type 1 in the left lower lobe, first diagnosed at 25 weeks’ gestational age. A lobectomy of the left lower lobe was performed 5 days postnatal. No other comorbidities were present, and no recurrent pulmonary infections appeared after surgery. At present, physical performance is reduced compared to his peers and breathing difficulties are noted during physical exertion.

**Fig. 4:**
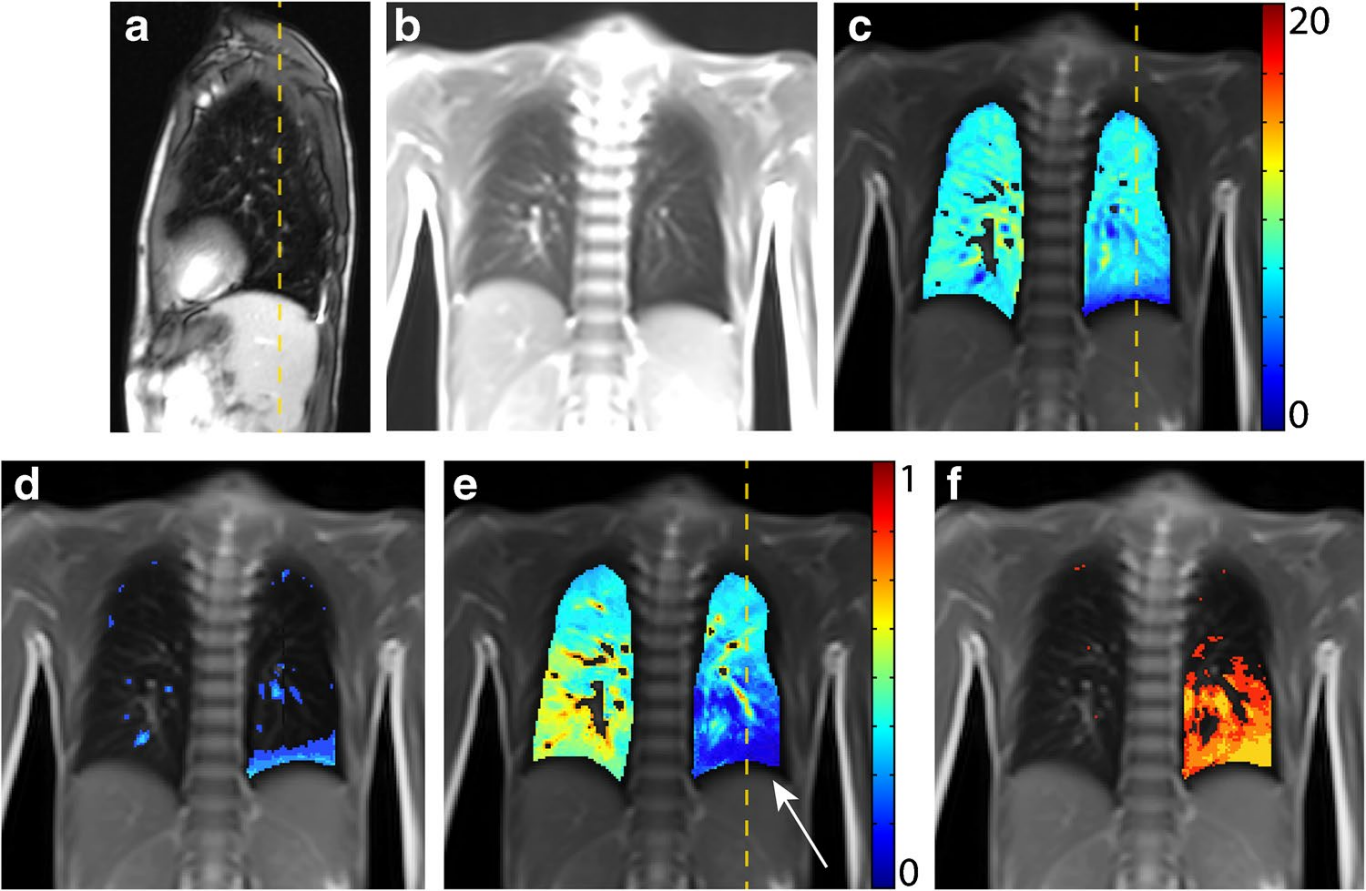
A 6-year-old boy following lobectomy of left lower lobe for CPAM type 1 with a normal lung clearance index of 7.32 turnovers. A sagittal T2-weighted image (**a**) (repetition time [TR]/echo time [TE] 3.3/1.3 ms) and (**b**) coronal T1/2-weighted image (TR/TE 1.5/07 ms) demonstrate a smaller volume of left lung and an expansion of the remaining left upper lobe into left lower thoracic cavity. **c-f** Coronal functional magnetic resonance imaging maps: (**c**) fractional ventilation, (**d**) fractional ventilation defect percentage (brighter blue areas represent reduced function), **(e)** relative perfusion and (**f)** relative perfusion defect maps (brighter orange areas represent reduced function) show a fractional ventilation defect percentage (13.4%) and a perfusion defect percentage (18.3%) within the norm. However, in the area of the former left lower lobe, which is now replaced by the upper left lobe, a locally reduced relative perfusion is visible in (**e**) (*white arrow*). *Yellow dashed lines* indicate plane levels. *CPAM* congenital pulmonary airway malformation

On structural MRI, the left lung is of reduced volume and appears slightly emphysematous. Additionally, asymmetrical diaphragm motility with slightly reduced motility on the left side are seen. Functional MRI shows an overall fractional ventilation and perfusion defect percentage within the norm (Fig. 4). Nevertheless, in the left lower thorax cavity, where the left upper lobe and lingula are present, a well-defined area with a fractional ventilation and perfusion defect is visible. The lung clearance index is within the norm, while pulmonary function tests show a mixed obstructive-restrictive disorder.

### Case 5

A 6-year-old girl with a CPAM type 1 in the left upper lobe, first diagnosed at 36 weeks’ gestational age. Surgical resection of segments I-III (left) by atypical stapler-resection was performed 8 days postnatal. No other comorbidities and an uneventful clinical history up to the present are reported.

**Fig. 5:**
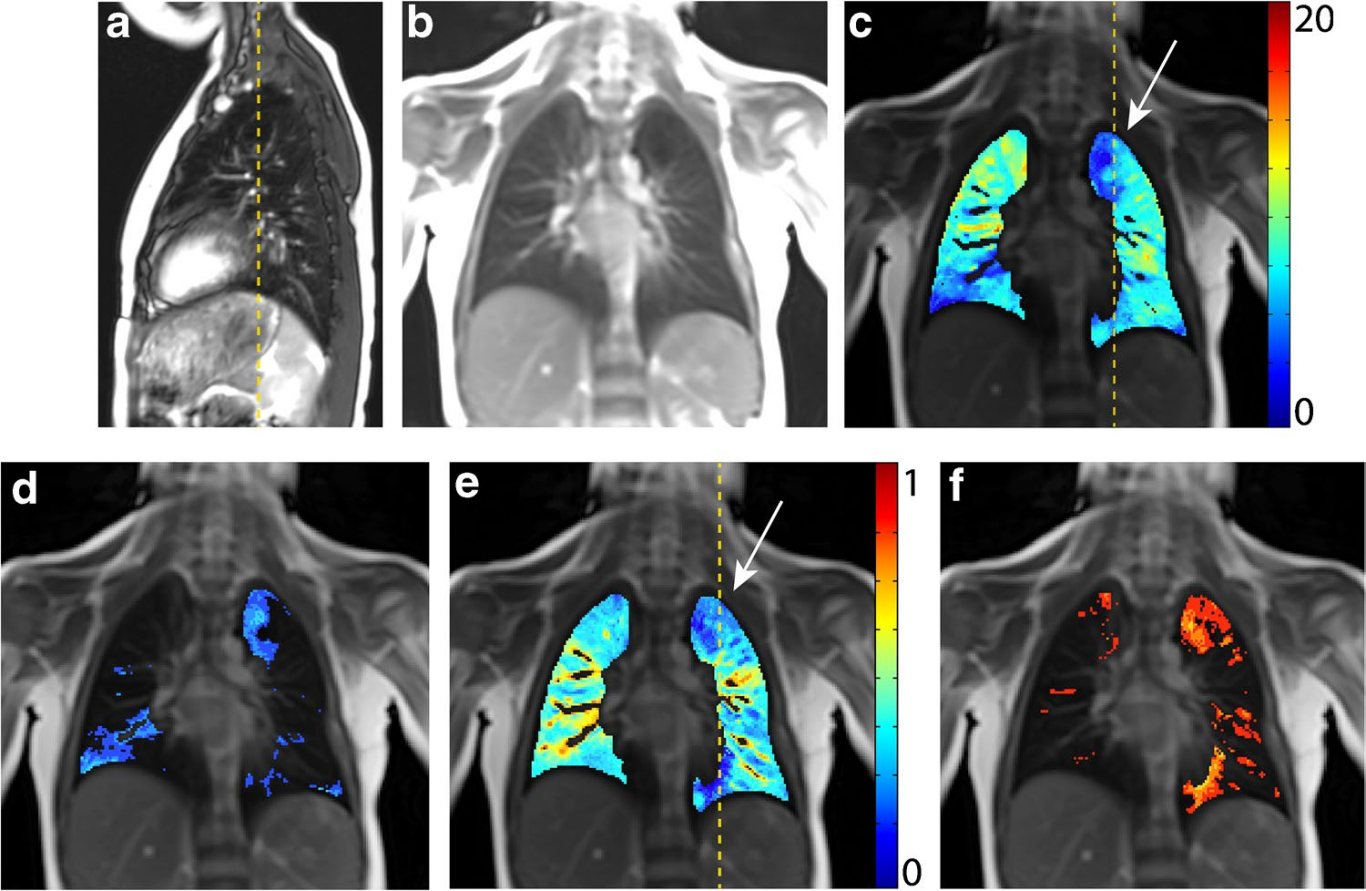
A 6-year-old girl following atypical stapler-resection of Seg I-III of the left lung for CPAM type 1 with a normal lung clearance index of 6.6 turnovers. A sagittal T2-weighted image (**a**) (repetition time [TR]/echo time [TE] 3.3/1.3 ms) and (**b**) coronal T1/2-weighted image (TR/TE 1.5/07 ms) show lung structures in the area of the former left lung segments I-III. No further abnormalities were visible. **c-f** Coronal functional magnetic resonance imaging maps: (**c**) fractional ventilation, (**d**) fractional ventilation defect percentage (brighter blue areas represent reduced function), **(e)** relative perfusion and (**f)** relative perfusion defect maps (brighter orange areas represent reduced function) show a fractional ventilation defect percentage (13.6%) and a perfusion defect percentage (14.5%) of the whole lung within the norm. However, in the left upper lobe, reduced fractional ventilation (**c**) and relative perfusion (**e**) are visible (*white arrow*). *Yellow dashed lines* indicate plane levels. *CPAM* congenital pulmonary airway malformation

Structural MRI presents no abnormalities, except for residual changes after surgery in the left upper lung and lung structures in the former segments I-III of the left lung. On the functional MRI, although the overall VDP and QDP are within normal range, there is a relative reduction of perfusion and fractional ventilation in the area of the former segments I-III (Fig. 5). Pulmonary function tests, as well as lung clearance index, show normal results.

## Discussion

In this pilot study, we present five children following lung resection for congenital lung malformations. In all patients, structural MRI showed that the remaining lung expands into the area of the resected lung. In this specific part of the lung, fractional ventilation and relative perfusion measured by functional MRI were impaired. In all other parts of the lung, fractional ventilation and perfusion were normal. In the three children in whom the local impairment of perfusion led to an increased overall perfusion defect percentage, the lung clearance index as measured by multiple breath washout was increased, indicating a ventilation inhomogeneity. Results of traditional pulmonary function tests, such as spirometry and body plethysmography, were heterogeneous, with obstruction, restriction and normal lung function.

While animal studies -- mostly using morphometric techniques -- demonstrate compensatory parenchymal growth (an increase in tissue as well as some remodeling) after lung resection, the assessment of compensatory lung growth in humans is largely based on pulmonary function testing [2, 29]. Here, a compensatory growth is thought to be present, when the measured lung volume parameters are larger than the volume expected from the amount of lung tissue resected. Distension of the remaining lung parenchyma on the other hand is assumed when the ratio of residual volume to total lung capacity is increased [2, 29, 30]. Interpretation is complicated by the fact that the results of different studies on long-term lung function outcomes in children following lung resection remain vary, depending on the patient group selected [1, 11–13, 31]. In line with these studies, normal as well as pathological results of pulmonary function testing were found in our pilot study. Therefore, assessing potential compensatory growth of the remaining lung after lung resection, based on conventional pulmonary function tests, is difficult.

**Fig. 6:**
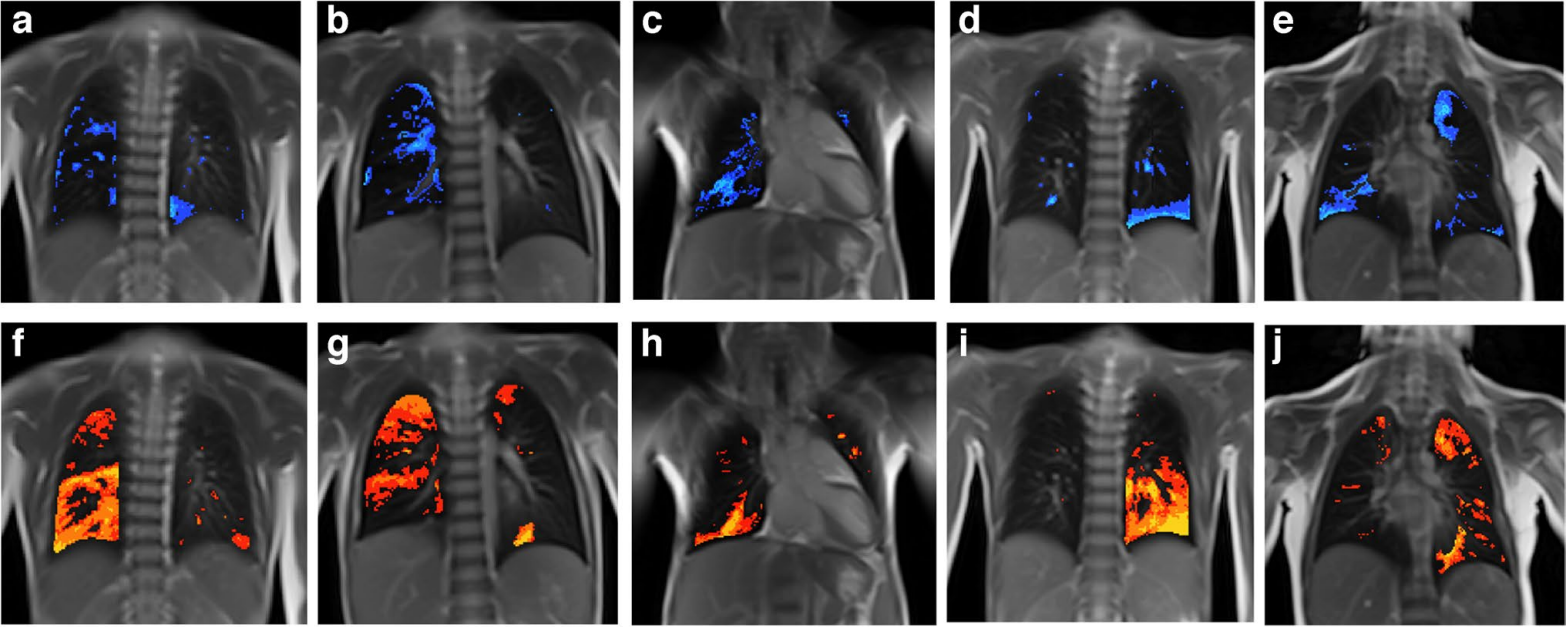
**a-j** Coronal functional magnetic resonance imaging maps with fractional ventilation defect maps in the upper row and perfusion defect maps in the lower row (brighter blue and orange areas represent reduced function). Case 1 (**a, f**) 7-year-old girl, case 2 (**b, g**) 5-year-old boy, case 3 (**c, h**) 10-year-old girl. Cases 1-3 after lobectomy of the right lower lobe. Case 4 (**d, i**) 6-year-old boy after lobectomy of the left lower lobe. Case 5 (**e, j**) 6-year-old girl after resection of left Seg I-III

Previous studies of the remnant lung after lobectomy in adult living lung transplant donors observed spirometry outcomes within the normal range and an ipsilateral increase in lung volume, as measured by CT. Authors concluded that physiological compensatory lung growth is possible [32]. However, it is unclear whether the tests can assess small airway function and overall ventilation homogeneity. In our pilot study, we observed normal spirometry outcomes, but have additionally investigated ventilation homogeneity by lung clearance index. A study investigating lung volumes, mechanics, and perfusion after lung resection, however, found reduced perfusion in the area of lung resection. This would support the idea that the residual lung]distends rather than undergoing compensatory growth [29, 33]. Considering the functional MRI images in our pilot study, the regional impairment of ventilation and perfusion (Figs. 1, 2, 3, 4, 5 and 6) seems to appear predominantly in region of the previously resected lung. This suggests that there is expansion, rather than fully functional compensatory growth, of the remaining lung [29, 33]. The situation in patients with congenital diaphragmatic hernia regarding lung expansion is comparable to patients following lung resection due to congenital lung malformations. Studies in congenital heart disease have shown impaired perfusion in the ipsilateral lung [34]. This is in line with our findings, with the difference being that in our patients the impairment of ventilation and perfusion is limited to small, local parts of the lung and thus does not result in overall impaired outcomes.

Lung clearance index can detect small airway disease even at an early stage, where pathological processes are already ongoing but not measurable by traditional spirometry. This has not only been demonstrated in patients with cystic fibrosis but also in smokers and patients with chronic obstructive pulmonary disease [35, 36]. In our patients with global perfusion over the upper limit of normal, an increased lung clearance index was seen. This leads to the assumption that in patients with distended lung following lung resection a small airway dysfunction is present, which is quantifiable by perfusion maps and lung clearance index. Therefore, congenital lung malformation resection in asymptomatic patients should be undertaken carefully, as the resection might also harm the remaining lung. Additionally, lung clearance index should be investigated in larger populations of patients following lung resection as a potential screening tool for referral for lung MRI.

All these findings suggest that the lung structures seen in the thoracic cavity region of the previously resected lung are not fully ventilated and perfused lung parenchyma. How the expanded and functionally impaired part of the lung is affected by recurring infections or other complications and if the risk of complications is the same as in children not following surgery for asymptomatic congenital lung malformations is unknown. This, however, could play a role in decision-making for managing asymptomatic congenital lung malformations. These pilot data encourage more systematic follow-up of children having lung resection and should prompt studies of larger, longitudinal cohorts.

Our pilot study has several strengths. First, we used novel methods that enabled us to assess functional impairment of ventilation and perfusion on a local level. This is not possible by commonly used lung function tests or conventional structural imaging. Further, we chose an age range for follow-up that allowed the lung to expand after surgery but was still within the period of active lung growth. The major limitation of this pilot study is the low number of patients and the heterogeneous clinical picture. Since this pilot study was limited to five patients, it was not possible to perform statistical analysis. Although a relevant influence of the mild comorbidities mentioned in two cases is unlikely, it cannot be ruled out. Additionally, lung function tests, such as spirometry and non-sedated lung MRI, are only feasible from 6 years of age onwards. It remains difficult to get a complete picture of lung development in earlier years. The application of ultrashort echo-time sequences to further assess lung parenchyma was not possible as this sequence type was not commercially available [37].

## Conclusion

The remaining lung after lung resection expands into the thoracic cavity region of the resected lung. Functional MRI shows that in this specific area, the lung does not function properly with regard to fractional ventilation and perfusion. These changes are not captured by conventional measures such as structural MRI and standard pulmonary function tests. Therefore, functional MRI is of additional value in assessing patients following lung resection for congenital lung malformations and should be investigated more systematically.

## Supplementary Information

ESM 1 (PDF 323 kb)
