# Supplementary figures and images for: School-age structural and functional MRI and lung function in children following lung resection for congenital lung malformation in infancy
Source: Pediatr Radiol. 2022 Mar 19;52(7):1255–65. doi: 10.1007/s00247-022-05317-7 (PMC9192451; doi:10.1007/s00247-022-05317-7)

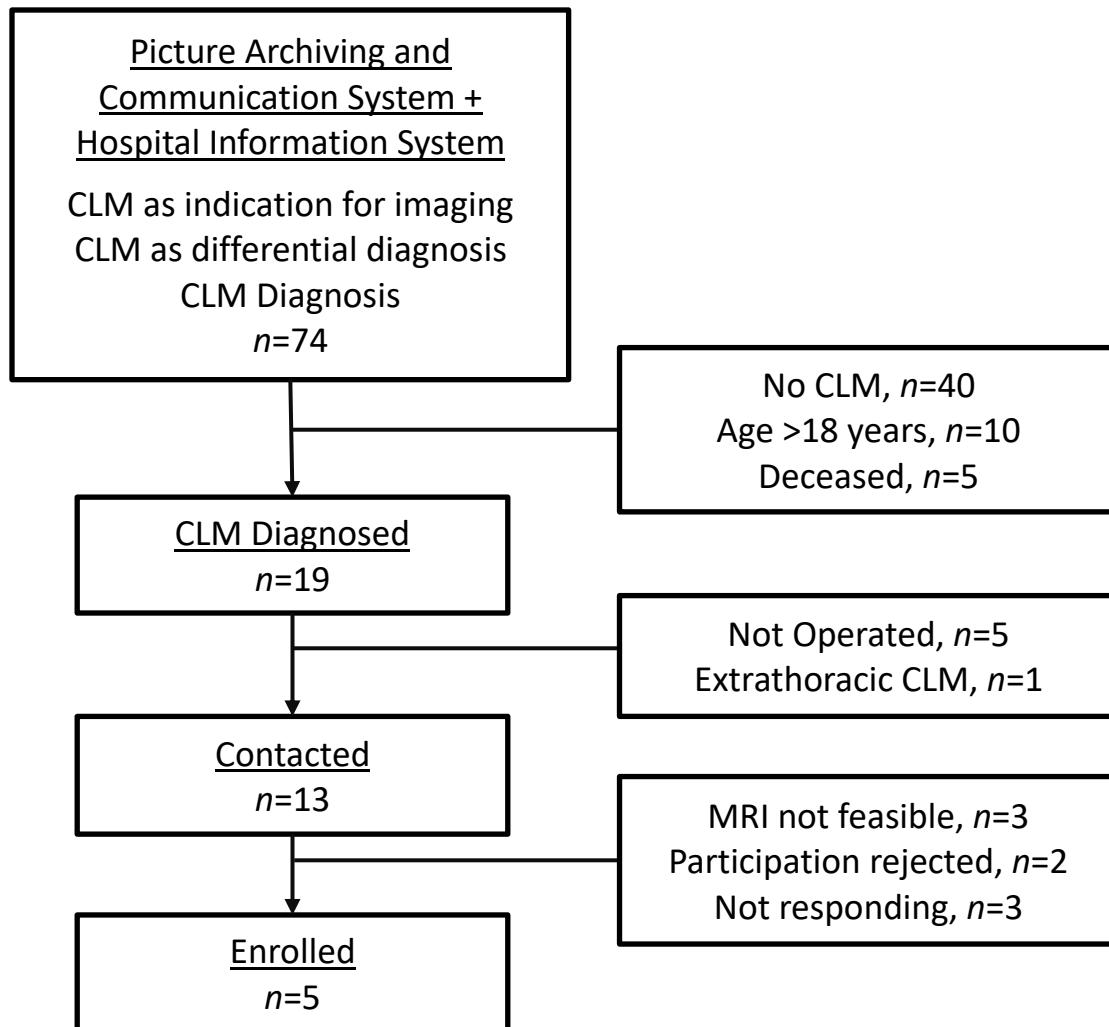

CLM: congenital lung malformation

Supplement: Supplementary file 1 — (PDF 323 kb) [file 247_2022_5317_MOESM1_ESM.pdf]
